# Dispensability of HPF1 for cellular removal of DNA single-strand breaks

**DOI:** 10.1093/nar/gkae708

**Published:** 2024-08-20

**Authors:** Kristyna Hrychova, Kamila Burdova, Zuzana Polackova, Despoina Giamaki, Beatrice Valtorta, Jan Brazina, Katerina Krejcikova, Barbora Kuttichova, Keith W Caldecott, Hana Hanzlikova

**Affiliations:** Laboratory of Genome Dynamics, Institute of Molecular Genetics of the Czech Academy of Sciences, Prague 4142 20, Czech Republic; Faculty of Science, Charles University in Prague, Prague 2128 43, Czech Republic; Laboratory of Genome Dynamics, Institute of Molecular Genetics of the Czech Academy of Sciences, Prague 4142 20, Czech Republic; Laboratory of Genome Dynamics, Institute of Molecular Genetics of the Czech Academy of Sciences, Prague 4142 20, Czech Republic; Institute of Animal Pathology, Vetsuisse Faculty, University of Bern, Bern 3012, Switzerland; Laboratory of Genome Dynamics, Institute of Molecular Genetics of the Czech Academy of Sciences, Prague 4142 20, Czech Republic; Faculty of Science, Charles University in Prague, Prague 2128 43, Czech Republic; Genome Damage and Stability Centre, University of Sussex, Falmer, Brighton BN1 9RQ, UK; Laboratory of Genome Dynamics, Institute of Molecular Genetics of the Czech Academy of Sciences, Prague 4142 20, Czech Republic; Laboratory of Genome Dynamics, Institute of Molecular Genetics of the Czech Academy of Sciences, Prague 4142 20, Czech Republic; Genome Damage and Stability Centre, University of Sussex, Falmer, Brighton BN1 9RQ, UK; Laboratory of Genome Dynamics, Institute of Molecular Genetics of the Czech Academy of Sciences, Prague 4142 20, Czech Republic; Institute of Animal Pathology, Vetsuisse Faculty, University of Bern, Bern 3012, Switzerland

## Abstract

In response to DNA damage, the histone PARylation factor 1 (HPF1) regulates PARP1/2 activity, facilitating serine ADP-ribosylation of chromatin-associated factors. While PARP1/2 are known for their role in DNA single-strand break repair (SSBR), the significance of HPF1 in this process remains unclear. Here, we investigated the impact of HPF1 deficiency on cellular survival and SSBR following exposure to various genotoxins. We found that HPF1 loss did not generally increase cellular sensitivity to agents that typically induce DNA single-strand breaks (SSBs) repaired by PARP1. SSBR kinetics in HPF1-deficient cells were largely unaffected, though its absence partially influenced the accumulation of SSB intermediates after exposure to specific genotoxins in certain cell lines, likely due to altered ADP-ribosylation of chromatin. Despite reduced serine mono-ADP-ribosylation, HPF1-deficient cells maintained robust poly-ADP-ribosylation at SSB sites, possibly reflecting PARP1 auto-poly-ADP-ribosylation at non-serine residues. Notably, poly-ADP-ribose chains were sufficient to recruit the DNA repair factor XRCC1, which may explain the relatively normal SSBR capacity in HPF1-deficient cells. These findings suggest that HPF1 and histone serine ADP-ribosylation are largely dispensable for PARP1-dependent SSBR in response to genotoxic stress, highlighting the complexity of mechanisms that maintain genomic stability and chromatin remodeling.

## Graphical Abstract

**Graphical Abstract:**
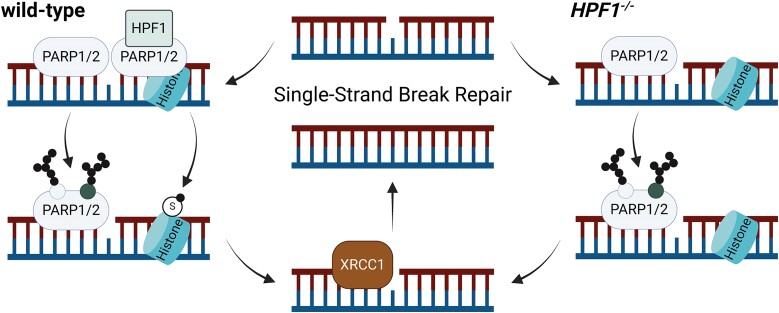

## Introduction

DNA single-strand breaks (SSBs) represent the most common DNA lesions encountered in cells, posing a substantial threat to both cell survival and genetic integrity (1). This is evident through increased occurrences of genetic deletion, embryonic lethality, and neurological disease observed when DNA single-strand break repair (SSBR) is compromised (2). SSBs arise from various sources, including oxidative attack of deoxyribose by reactive oxygen species (ROS), intermediates of the excision repair of damaged DNA bases (known as DNA base excision repair; BER) or ribonucleotides (known as ribonucleotide excision repair; RER), as abortive products of topoisomerase 1 (TOP1) activity, and as unligated Okazaki fragment intermediates during DNA replication (3–8). Upon their occurrence, SSBs are rapidly detected by DNA damage-inducible poly-ADP-ribose polymerases (PARPs) (9,10). PARP1, the founding member of the PARP family, is typically responsible for more than 80% of ADP-ribose synthesis within cells (10–12). PARP1 and PARP2 modify themselves and neighbouring proteins, generating mono- and poly-ADP-ribose molecules, thereby facilitating chromatin accessibility and recruiting the XRCC1 scaffold protein to sites of DNA damage (9,13–15). XRCC1 assembles DNA repair protein complexes containing SSBR enzymes such as DNA polymerase β (POLβ), aprataxin (APTX), polynucleotide kinase-phosphatase (PNKP), DNA ligase 3 (LIG3) or tyrosyl-DNA phosphodiesterase 1 (TDP1) (2). ADP-ribosylation is rapidly reversed by glycohydrolases, enabling the reactivation of automodified PARPs (16). Poly-ADP-ribose glycohydrolase (PARG) is the most efficient of these enzymes, but it does not cleave the terminal (protein-proximal) ADP-ribose moiety (17). The removal of protein-proximal ADP-ribose is ensured by mono-ADP-ribose glycohydrolases ARH3 and TARG, which eliminate mono-ADP-ribose from serine and glutamate/aspartate residues, respectively (18–20).

Recently, the DNA damage-responsive protein histone PARylation factor-1 (HPF1) was identified as a binding partner of PARPs, particularly PARP1 and PARP2 (21). Following DNA damage, HPF1 binds PARP1/2 at the site of the break, regulating their activity and conferring their substrate preference for serine residues (22–24). Importantly, following exposure to hydrogen peroxide, serine is a major residue modified by ADP-ribosylation, and the loss of HPF1 leads to decreased level of ADP-ribosylation, primarily on histones (25,26). Histones undergo extensive modifications through other post-translational mechanisms, including acetylation, methylation, phosphorylation, and ubiquitination. Recent quantitative proteomic analysis based on mass spectrometry revealed that histones are co-modified with serine APD-ribosylation and other post-translational modifications (PMTs) after exposure to hydrogen peroxide (23). Moreover, it has been demonstrated that the acetylation of H3K9 and the ADP-ribosylation of H3S10 are mutually exclusive, suggesting an intricate interplay between serine APD-ribosylation and other histone PTMs, potentially impacting chromatin remodeling and transcription regulation during DNA break repair (27).

Despite these insights, the specific roles for HPF1-dependent ADP-ribosylation within the cellular context remain unclear. Based on previous findings (25,28), it was proposed that HPF1-dependent ADP-ribosylation is crucial for proper DNA repair following DNA damage. However, the actual impact on the rate of SSB induction and the efficiency of SSBR in HPF1-deficient cells after DNA damage have not yet been explored. We therefore investigated the role of histone HPF1-dependent ADP-ribosylation at DNA damage sites using CRISPR/Cas9 gene-edited HPF1- and/or ARH3-deficient cells. Given the established impact of ADP-ribose metabolism on SSBR, it was anticipated that HPF1-deficient cells would exhibit DNA repair defects and cellular sensitivity to DNA damage. Surprisingly, our data suggest that HPF1 is largely dispensable for the repair of a broad range of SSBs in response to various DNA-damaging agents. While near-normal levels of poly-ADP-ribosylation, likely responsible for the recruitment of DNA repair factors, are retained at the chromatin in *HPF1^−/−^* cells following DNA damage, levels of mono-ADP-ribosylation are significantly reduced or abolished. Interestingly, our previous work indicated that the loss of ARH3, responsible for the cleavage of ADP-ribose from histone serine residues following DNA damage, also does not affect SSBR (18). Collectively, these findings suggest that serine histone mono-ADP-ribosylation is largely dispensable for DNA single-strand break repair in human cells and may serve additional role/s.

## Materials and methods

### Cell lines and culture

Human hTERT RPE-1 (denoted RPE-1) and U2OS cell lines (Supplementary Table S1) were grown in high glucose DMEM (Sigma-Aldrich) and DMEM/F-12 (Gibco), respectively, supplemented with 10% serum and the antibiotics penicillin/streptomycin at 37°C, 5% CO_2_ and 5% oxygen. The cells were regularly tested for mycoplasma contamination and were found to be clean. Wild-type RPE-1 and the gene-edited *PARP1^−/−^* (clone #G7), *PARP2^−/−^* (clone #A1), *PARP1^−/−^*/ *PARP2^−/−^* (clone #E6) and *XRCC1^−/−^* (clone #3) cell lines and wild-type U2OS (I) and *XRCC1^−/−^* (clone #2) cells have been described previously (13,29,30). Wild-type U2OS (II) and their gene edited cell lines *HPF1^−/−^* (II-cl.1) and *ARH3^−/−^* (clone #48) were a gift from Ivan Ahel at the University of Oxford (19,21). To induce DNA single-strand breaks, cells were exposed to the specified dose of hydrogen peroxide (H_2_O_2_), camptothecin (CPT) or methyl methansulfonate (MMS). The treatment was conducted either on ice in serum-free media, followed by incubation with full media at 37°C, or by direct incubation in full media at 37°C for the indicated periods. All genotoxins were obtained from Sigma-Aldrich (H1009, 129925 and C9911). H_2_O_2_ and MMS were dissolved directly in the media, while a 10 mM CPT solution was prepared using DMSO. Final concentrations are specified in the figure legends. The PARP inhibitor Olaparib (S1060; purchased from Selleck Chemicals) was used at a final concentration 10 μM.

### Generation of CRISPR/Cas9 deleted cell lines

CRISPR/Cas9 gene edited U2OS (I) or RPE-1 cell lines generated in this study were prepared using RNA guides listed in Supplementary Table S2. For generation of *HPF1^−/−^* and *ARH3^−/−^* knockout cell lines, cells were transfected with Cas9-GFP plasmid (Addgene 48138) and plasmids encoding HPF1 or ARH3 gRNA#1 and gRNA#2 using Lipofectamine LTX (Invitrogen) in 1:1:1 ratio. Transfected cells were sorted (BD FACSMelody) for GFP positive cells two days after transfection and seeded into 96-wells plates for clonal selection. Clones were analyzed by western blotting once at sufficient cell density. Gene editing was confirmed by Sanger sequencing. Genomic DNA from clones was isolated (Qiagen; DNeasy) and part of HPF1 or ARH3 gene was amplified by PCR (Phusion polymerase, NEB) and PCR products were subcloned into pCR®2.1-TOPO® using TOPO cloning kit (Invitrogen). Plasmid DNA was isolated (SmartPure Plasmid Kit; Eurogentec) and sequenced using M13R primer (Eurofins Genomics). Verified clones were selected for further experiments; *HPF1^−/−^* U2OS (clone I-#5; out-of-frame deletion in all alleles, and clone I-#8; out-of-frame deletion in all alleles) *HPF1^−/−^* RPE-1 (clone #5; out-of-frame deletion in both alleles, and clone #10; out-of-frame deletion and out-of-frame insertion) and *ARH3^−/−^* RPE-1 (clone #1; out-of-frame deletion in both alleles). *HPF1^−/−^*/*ARH3^−/−^* U2OS cells (clone #D; out-of-frame deletion in both alleles) were generated by targeting HPF1 in *ARH3^−/−^* U2OS (clone #48).

For generation of *PARP1^−/−^* and *PARP2^−/−^* knockout cell lines, two hundred thousand cells were electroporated with Cas9 RNPs (120 pmol crRNA, 120 pmol tracrRNA and 100 pmol His-Cas9-GFP in Cas9 buffer; 20 mM HEPES pH 7.5, 150 mM NaCl, 2 mM MgCl_2_, 1 mM TCEP) and the appropriate guide RNA construct using a NEON Transfection System (Invitrogen) with a 10 μl tip using 1230 V/10 and width/4 pulses. The cells were reseeded to 96-well plates three days after electroporation, and single clones were picked, analyzed by western blotting, and sequenced. Verified clones were selected for further experiments; *PARP1^−/−^* U2OS (clone #15) and *PARP2^−/−^* U2OS (clone #5). The generation of the *PARP1^−/−^*/*PARP2^−/−^* U2OS cell line (clone #5) was carried out by targeting *PARP2* in *PARP1^−/−^* (clone #15) U2OS cells. *HPF1^−/−^*/*PARP1^−/−^* RPE-1 cells (clone #14 and #21) and *HPF1^−/−^*/*PARP2^−/−^* (clone #3 and #7) were generated by targeting PARP1 or PARP2, respectively, in *HPF1^−/−^* RPE-1 (clone #5).

### Alkaline comet assay

Trypsinized U2OS or RPE-1 cells and gene-edited derivatives were washed and treated with different genotoxins as indicated in the text. After incubation, cells were washed once with ice-cold PBS, resuspended in cold PBS, and spread onto agarose-coated slides by mixing with a low-melting agarose solution in a 1:1 ratio. Cells were lysed in a lysis buffer (2.5 M NaCl, 100 mM EDTA, 10 mM Tris, adjusted by NaOH to pH 10, 1% DMSO, 1% Triton X-100) for 1 hour at 4°C, followed by unwinding in an alkaline running buffer (50 mM NaOH, 1 mM EDTA, 1% DMSO, pH > 13) for 45 min. Slides were placed in a comet electrophoresis tank (Thermo), shielded from light, in a cold room at 12–15 V for 25 min. Using *Method I*, slides were subsequently neutralized using 0.4 M Tris pH 7.4 for at least 15 min, followed by staining with SYBR Green (Sigma-Aldrich) diluted in PBS containing antifade (40 μg/ml phenylenediamine dihydrochloride, Fisher) for 15 min in the dark at room temperature (RT). The comets were imaged and scored using Comet Assay IV software (Perceptive Instruments). Alternatively, using *Method II*, slides were neutralized using 0.4 M Tris pH 7.4 for 15 min, followed by staining with SYBR Green (Sigma-Aldrich) diluted in TE buffer for 15 min in the dark at room temperature (RT). Slides were washed three times with dH_2_O and dried. Images were acquired using an Olympus IX81 microscope equipped with the ScanR high-content module and a UPLSAPO 10x/0.4 objective and analysed using CometScore 2.0 (Tritek). A minimum of 50 cells were analysed per condition.

### Clonogenic survival assay

U2OS or RPE-1 cells and gene-edited derivatives were trypsinized, counted, and 300–500 of cells were plated in 10 cm dishes. The cells were allowed to attach for 4 hours in the incubator. Subsequently, the cells were treated with the indicated dose of MMS in full media, or H_2_O_2_ in serum-free media at RT for 15 and 10 min, respectively. To inactivate H_2_O_2_, serum was added to the plates to reach a final concentration of 10%. For MMS treatment, cells were washed twice with PBS, and full medium was added to the plates. CPT treatment was performed continuously in full media. The cells were then left to form colonies for 10–12 days before being fixed in 96% ethanol and stained with crystal violet solution (0.05% crystal violet, 4% formaldehyde, 1% methanol). Colonies containing more than 50 cells were counted, and the relative clonogenic potential was calculated. The surviving fraction at each dose was calculated by dividing the average number of colonies in the treated dishes by the average number in the untreated dishes.

### Indirect immunofluorescence and microscopy

Cells were seeded onto coverslips and treated the following day as described in the text. Subsequently, they were washed with cold PBS, pre-extracted using extraction buffer (25 mM HEPES pH 7.4, 50 mM NaCl, 1 mM EDTA, 3 mM MgCl_2_, 0.3 M sucrose, 0.5% Triton X-100) for 5 min on ice, and fixed for 15 min on ice using cold 4% formaldehyde (VWR). After fixation, cells were permeabilized with 0.5% Triton X-100 in PBS for 5 min at RT, blocked with 5% BSA in PBS for 30 min, and incubated with the primary antibody (listed in Supplementary Table S3), appropriately diluted in BSA, for 1h at RT. After three washes with PBS, cells were incubated with the secondary antibody (listed in Supplementary Table S3) in BSA for 40 min. Subsequently, coverslips were washed three times with PBS, incubated with DAPI in PBS for 5 min, and briefly washed with distilled water. Once dried, coverslips were mounted using Vectashield. Images were captured using an Olympus IX81 microscope equipped with the ScanR high-content module and a UPLXAPO 20x/0.8 objective. Nuclei were segmented based on DAPI signals using the ScanR analysis software (Olympus). The acquired data were analysed using FlowJo (TreeStar) software. A minimum of 1000 nuclei were analysed for each condition.

### Cell transfection and laser-microirradiation

U2OS cells were seeded into glass-bottom dishes (Cellvis) and transfected with the mRFP-XRCC1 construct using jetPRIME (Polyplus) (14). RPE-1 cells were electroporeted with the mRFP-XRCC1 construct using a NEON Transfection System (Invitrogen) with a 10 μl tip using 1350 V, 20 ms width/2 pulses, and then seeded into glass-bottom dishes (Cellvis). Cells were pre-sensitized by incubation with 10 μM BrdU (Sigma-Aldrich) 24 h after transfection, and the laser-microirradiation experiments were conducted 48 hours after transfection. Before microirradiation, the medium was changed to DMEM without phenol red (Gibco) supplemented with 10% serum and penicillin/streptomycin (Gibco). Laser-microirradiation experiments were performed using Leica Stellaris 8 confocal microscope, controlled by LAS X software and equipped with an incubation chamber set at 37°C and 5% CO_2_ for live-cell imaging. Cells were microirradiated with a 405 nm UV-laser at maximum power, and time-lapse images were acquired at the indicated times after microirradiation using 40×/1.3 oil objective as previously described (31).

### hPARG purification and *in situ* reactions

Maltose binding protein (MBP) and cDNA of human PARG sequence was cloned into the pET28a vector with His-tag at the C-terminus. Protein was expressed in BL21 (DE3) Gold strain with 0.1 mM IPTG induction at 16°C overnight. Bacterial pellet was resuspended in ice cold lysis buffer (10 mM Tris pH 7.5, 0.5 M NaCl, 0.1% NP-40, 10 mM imidazole, 1 mM DTT, 10% glycerol), sonicated and centrifuged at 20 000 g for 30 min at 4°C. Soluble fraction was incubated with Ni-NTA beads for 1 hour at 4°C, beads were washed with lysis buffer, wash buffer (10 mM Tris pH 7.5, 0.2 M NaCl, 30 mM imidazole, 1 mM DTT, 10% glycerol) and protein was eluted with elution buffer (10 mM Tris pH 7.5, 0.5 M NaCl, 500 mM imidazole, 1 mM DTT, 10% glycerol). Eluted protein was diluted and applied to amylose beads, incubated for 45 min on ice, washed with 10 mM Tris pH 7.5, 200 mM NaCl, 10% glycerol and eluted with 25 mM maltose in wash buffer. Final protein was concentrated using Centricon with 3 kDa cut-off membrane. For the *in-situ* reactions, fixed cells on coverslips, after the blocking step with 5% BSA, were incubated with 200 nM hPARG in 10 mM Tris pH 7.5, 100 mM NaCl, 2 mM MgCl_2_ 1 h at 37°C. Subsequently, the coverslips were washed with PBS supplemented with 0.5 M NaCl and PBS, before incubation with the indicated antibodies.

### Production of recombinant iAf1521 antibody

The recombinant iAf1521 (improved Af1521) antibody was generated by preforming site-directed mutagenesis of K35E and Y145R, key mutations for ADP-ribose binding improvement (32), using QuikChange Lightning Multi Site-Directed Mutagenesis Kit (Agilent Technologies) in the pET28a vector, which contained a His-tagged rabbit Fc chain fused with the Af1521 domain. The production and purification of the iAF1521 antibody followed previously established protocol (33). To validate the binding properties of the iAF1521 antibody, we used *ARH3^−/−^* cell lines and PARG inhibitor (PARGi) treatment and compared the results with the original wild-type Af1521. We observed an increased signal in *ARH3^−/−^* cell lines, which remained unaffected by hPARG *in situ* treatment. Additionally, there was an increased signal in wild-type cells after PARGi treatment, which was sensitive to hPARG *in situ* treatment (Supplementary Figure S1D). This confirmed that the iAf1521 antibody binds to both to mono- (MAR) and poly-ADP-ribose (PAR). Furthermore, in comparison between the signal of iAf1521 and commercial mono-ADP-ribose-specific antibodies on western blot analysis using lysates from wild-type and *ARH3^−/−^* cells, we noted that iAf521 exhibited broader MAR detection properties (Supplementary Figure S1C). Pull-down experiments of ADP-ribosylated proteins from lysate of wild-type and *ARH3^−/−^* cells were performed as previously described to further compare the ADP-ribose biding properties of wild-type and non-binding G42E mutant Af1521, previously published eAf1521, and the generated iAf1521 (Supplementary Figure S1B) (32).

### SDS-PAGE and western blotting

Whole cell extracts were obtained by lysing cells washed with PBS in Laemmli sample buffer (2% SDS, 10% glycerol, 50 mM Tris–HCl pH 6.8), followed by sonication. To determine the sensitivity of ADP-ribosylations to hydroxylamine, cells were lysed in 10 mM HEPES pH 8.0, 2 mM MgCl_2_, 1% SDS buffer containing benzonase, and treated with hydroxylamine as previously described (25). Protein concentration was determined using BCA kit (Thermo); samples were adjusted, and bromphenol blue and DTT were added. Boiled samples were resolved by Tris-Glycine or Bis–Tris SDS-PAGE, and proteins were transferred onto nitrocellulose membranes. The membranes were initially stained with Ponceau S solution, destained, blocked with 5% milk in PBS-T, and subsequently incubated with primary antibody (listed in Supplementary Table S3) dilutions overnight at 4°C. The following day, the membranes were washed with PBS-T, incubated with corresponding HRP-coupled secondary antibodies (listed in Supplementary Table S3), washed again, and then developed (Optimax, Protec) using an ECL substrate (GE) and light-sensitive films (AGFA).

### Chromatin fractionation

Cells were trypsinized, treated with 2 mM hydrogen peroxide in full media for 20 min at 37°C in a water bath, then washed with cold PBS, resuspended in a lysis buffer (25 mM HEPES pH 7.4, 150 mM NaCl, 1 mM EDTA, 3 mM MgCl_2_, 0.3 M sucrose, 0.5% Triton X-100) supplemented with protease inhibitors (Roche), and incubated on ice for 15 min. At this point, whole cell (WC) extract samples were collected. The chromatin was separated from soluble fraction by centrifugation at 20 000 g 15 min at 4°C. The chromatin pellet was subjected to three washes with the lysis buffer, then resuspended in Laemmli sample buffer, boiled, and subsequently sonicated.

### Statistical analysis

Statistical analysis was conducted using GraphPad Prism v9 (GraphPad Software) to assess the significance among experimental replicates. All data are presented as the mean ± SD from a minimum of three independent biological replicates. A two-tailed unpaired Student's t-test or ANOVA were employed to analyse the experimental data. Experimental outcomes resulting in a *P-*value <0.05 were considered statistically significant. Symbols such as one asterisk (*), two asterisks (**), three asterisks (***) and four asterisks (****) were used to indicated statistical significance levels, denoting *P-*values of <0.05, 0.01, 0.001 and 0.0001, respectively.

## Results

### HPF1 is largely dispensable for cell survival in response to SSBs

**Figure 1.**
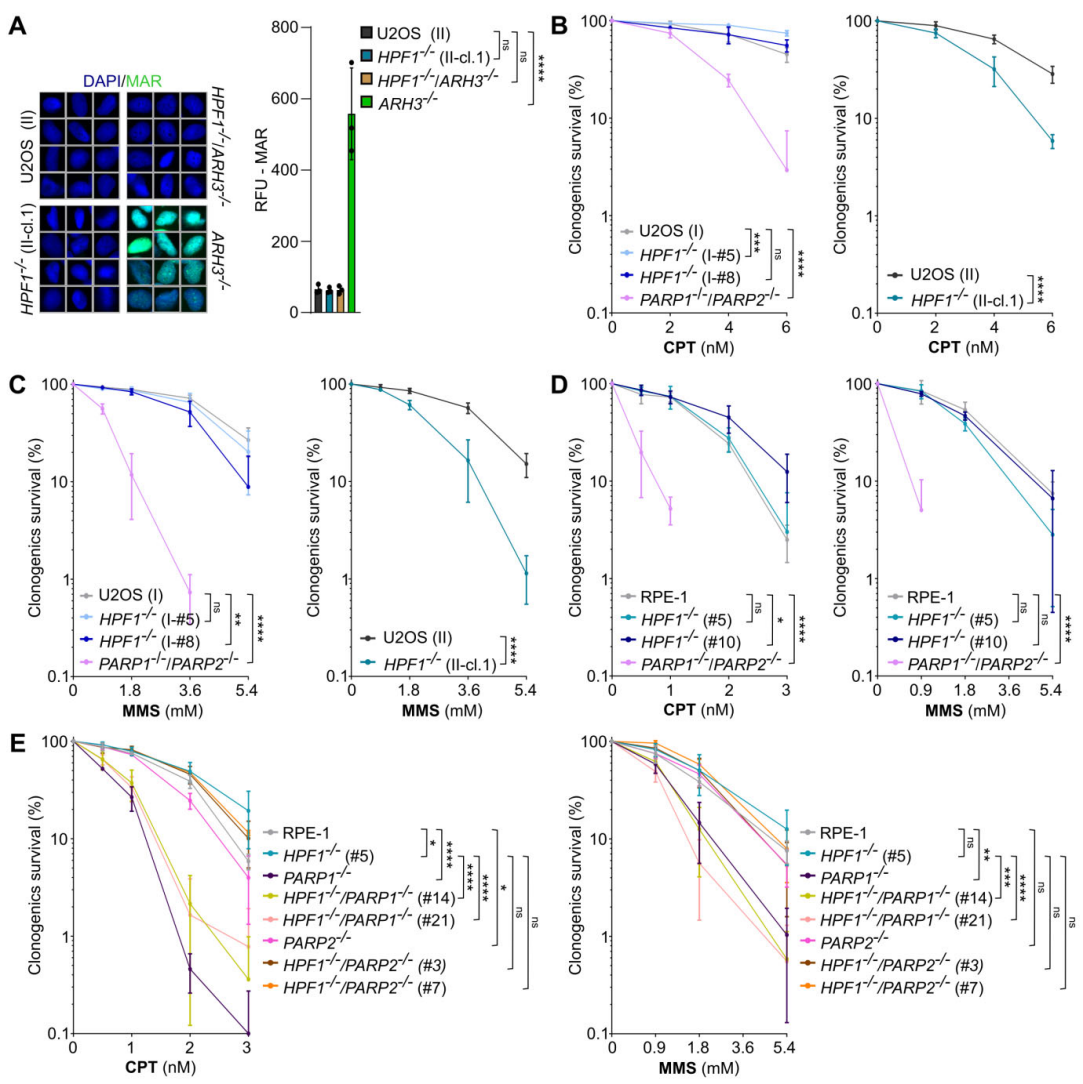
HPF1 is largely dispensable for cell survival in response to DNA single-strand breaks. (**A**) Immunofluorescence analysis of endogenous mono-ADP-ribosylation levels detected by a specific mono-ADP-ribose binding reagent (MAR 205) in U2OS (II) wild-type, *HPF1^−/−^* (II-cl.1), *ARH3^−/−^/HPF1^−/−^* (clone #D) and *ARH3^−/−^* (clone #48) cells. Representative ScanR images and quantifications are shown, RFU - relative fluorescence units. Data are the mean (±SD) of three independent experiments. Statistical analysis (one-way analysis of variance) is shown (ns – not significant, ****P < 0.0001). (**B**, **C**) Clonogenic survival assay in U2OS (I) wild-type, *HPF1^−/−^* (clone I-#5 and I-#8), *PARP1^−/−^/PARP2^−/−^* (clone #5) and U2OS (II) wild-type and *HPF1^−/−^* (II-cl.1) cells in response to treatment with indicated doses of CPT and MMS for 15 min at RT. Data represent the mean (±SD) of three independent experiments. Statistical analysis (two-way analysis of variance) is shown (ns – not significant, **P < 0.01, ***P < 0.001, ****P < 0.0001). (**D, E**) Clonogenic survival assay in RPE-1 wild-type, *HPF1^−/−^* (clone #5 and #10), *PARP1^−/−^/PARP2^−/−^* (clone #E6), and *PARP1^−/−^* (clone #G7), *PARP2^−/−^* (clone #A1), *HPF1^−/−^/PARP1^−/−^* (clone #14 and #21) and *HPF1^−/−^/PARP2^−/−^* (clone #3 and #7) cells in response to treatment with indicated doses of CPT and MMS for 15 minutes at RT. Data represent the mean (±SD) of three independent experiments. Statistical analysis (two-way analysis of variance) is shown (ns – not significant, *P < 0.05, **P < 0.01, ***P < 0.001, ****P < 0.0001).

To explore the significance of serine ADP-ribosylation in DNA repair within the cellular context, we employed human cell lines in which *HPF1* and/or *ARH3* were deleted using CRISPR/Cas9-mediated genome editing (Supplementary Figure S1A). Utilizing our newly generated ADP-ribose binding reagent (iAf1521) that efficiently recognizes both mono-ADP-ribose (MAR) and poly-ADP-ribose (PAR), along with specific antibodies targeting MAR and PAR, we observed the accumulation of endogenous MAR, but not PAR, in the chromatin of ARH3-deficient cells, in the absence of treatment with genotoxins (Supplementary Figure S1B-E). Importantly, most of this mono-ADP-ribosylation was absent from the chromatin of *HPF1^−/−^/ARH3^−/−^* cells, confirming that it reflected HPF1-dependent serine ADP-ribosylation (Figure 1A). The accumulation of endogenous HPF1-dependent serine ADP-ribosylation is consistent with previous findings that serine ADP-ribosylation is the prevalent type of ADP-ribosylation, particularly following DNA damage (25,34,35). To assess the importance of serine ADP-ribosylation for cellular resistance to DNA damage, we conducted clonogenic cell survival assays. We compared the sensitivity of generated U2OS cell lines to hydrogen peroxide (H_2_O_2_), camptothecin (CPT), and methyl methanesulfonate (MMS) which are genotoxins that collectively induce a range of DNA single-strand breaks (SSBs) requiring PARP1 and/or PARP2 activity for their repair. The *HPF1^−/−^* U2OS clones were not hypersensitive to H_2_O_2_, and additionally, the deletion of ARH3, which removes HPF1-mediated serine ADP-ribosylation from proteins, did not increase cellular sensitivity to H_2_O_2_, even if deleted in conjunction with HPF1 (Supplementary Figure S2A). We detected a mild sensitivity in certain *HPF1^−/−^* clones to CPT and MMS; however, the observed cellular sensitivity was moderate compared to the substantial sensitivity in *PARP1^−/−^/PARP2^−/−^* U2OS cells (Figure 1B and C), a finding consistent with published data (21,36).

To extend these experiments and delve deeper into the importance of serine ADP-ribosylation, we generated and employed additional CRISPR/Cas9-edited cell lines, specifically HPF1-deficient human diploid hTERT RPE-1 cells (denoted RPE-1) (Supplementary Figure S1A). Notably, we did not detect increased cellular sensitivity to CPT and MMS in *HPF1^−/−^* RPE-1 cells (Figure 1D). This was in contrast to *PARP1^−/−^/PARP2^−/−^* RPE-1 cells, which, as expected, exhibited significant hypersensitivity. Additionally, to demonstrate the importance of PARP1 for cell survival following DNA damage in HPF1-deficient cells, we conducted similar clonogenic experiments in *HPF1^−/−^/PARP1^−/−^* and *HPF1^−/−^/PARP2^−/−^* RPE-1 cells (Figure 1E). Taken together, our data strongly suggest that HPF1-dependent serine ADP-ribosylation plays a negligible role in maintaining cellular resistance to DNA-damaging agents, such as H_2_O_2_, CPT and MMS. Moreover, they indicate that PARP1, even in the absence of HPF1, is sufficient to govern DNA repair processes in these cells.

### HPF1 is not required for DNA single-strand break repair

**Figure 2.**
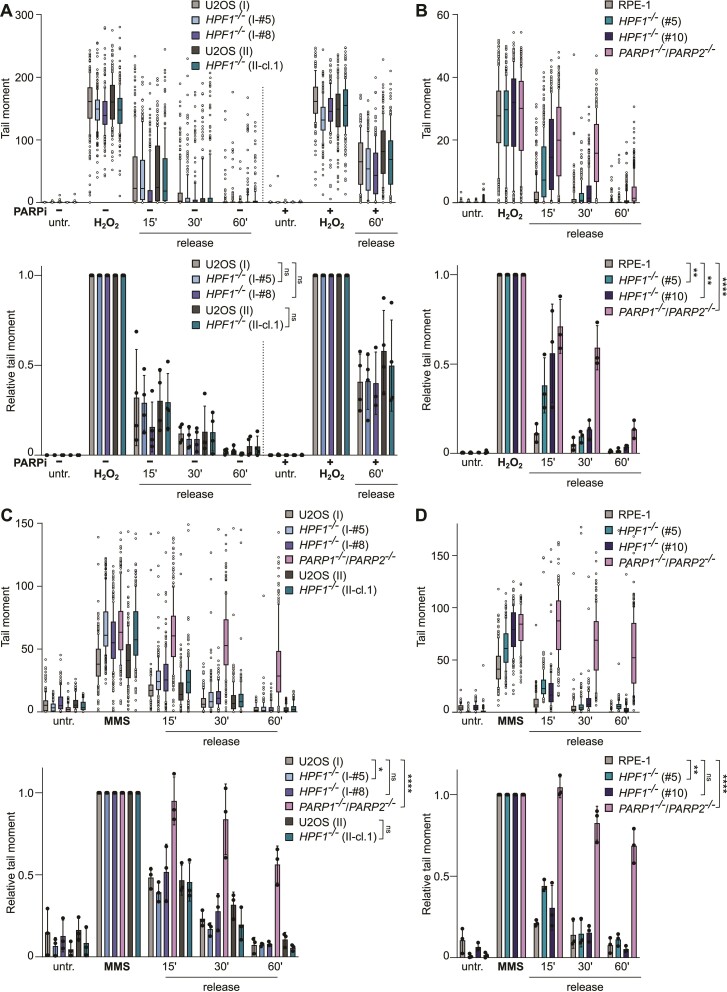
Impact of HPF1 deficiency on DNA single-strand break repair. (**A**) DNA strand breaks quantified by alkaline comet assays (*Methods II*) in U2OS (I) wild-type, *HPF1^−/−^* (clone I-#5 and I-#8), and U2OS (II) wild-type and *HPF1^−/−^* (II-cl.1), cells before and after 100 μM H_2_O_2_ treatment in serum-free media for 10 min on ice, followed by incubation at 37°C in full media (release time), in the presence or absence of 10 μM PARPi. The individual comet tail moments of cells combined from three to four independent experiments are plotted (*the upper chart*). A minimum of 50 cells were analysed per sample in each of the experiments. The normalized data are shown (*the lower chart*) and represent the relative mean (±SD) of three to four independent experiments. Statistical analysis (two-way analysis of variance) is shown (ns, not significant). (**B**) Alkaline comet assay analysis (*Methods I*) in RPE-1 wild-type, *HPF1^−/−^* (clone #5 and #10), and *PARP1^−/−^/PARP2^−/−^* (clone #E6) cells before and after 50 μM H_2_O_2_ treatment in serum-free media for 10 min on ice, followed by incubation at 37°C in full media (release time). The individual comet tail moments of cells combined from three independent experiments are plotted (*the upper chart*). A minimum of 50 cells were analysed per sample in each of the experiments. The normalized data are shown (*the lower chart*) and represent the relative mean (±SD) of three independent experiments. Statistical analysis (two-way analysis of variance) is shown (**P < 0.01, ****P < 0.0001). (**C, D**) Alkaline comet assay analysis (*Methods II*) in U2OS (**C**) or RPE-1 (**D**) clones, as indicated, before or after the treatment with 0.9 mM MMS for 15 min at 37°C, followed by incubation at 37°C after MMS wash (release time). The individual comet tail moments of cells combined from three independent experiments are plotted (*the upper charts*). A minimum of 50 cells were analysed per sample in each of the experiments. The normalized data are shown (*the lower charts*) and represent the relative mean (±SD) of three independent experiments. Statistical analysis (two-way analysis of variance) is shown (ns – not significant, *P < 0.05, **P < 0.01, ****P < 0.0001).

To gain a deeper insight into the role of HPF1 in SSBR, we conducted alkaline comet assays to measure the induction and repair of SSBs in wild-type, HPF1*-*deficient and PARP1/2-deficient cells after exposure to H_2_O_2_, CPT and MMS. As expected, PARP1/2-deficient U2OS and RPE-1 cells showed reduced SSBR rates in response to all three genotoxins (Figure 2B–D, Supplementary Figure S2B and C). In contrast, *HPF1*^−/−^ U2OS cell lines did not exhibit reduced rates of DNA strand break repair following treatment with H_2_O_2_, a physiologically relevant source of oxidative stress that primarily introduce (>99%) SSBs (Figure 2A). Notably, a pronounced defect in SSBR upon H_2_O_2_ treatment was evident in HPF1-deficient U2OS cells only following PARP inhibition. Interestingly, *HPF1^−/−^* RPE-1 cells showed a slight delay in DNA strand break repair at early time points (∼15 min) post-H_2_O_2_ treatment, but this delay was not observed 30–60 min after exposure. This contrasts with *PARP1^−/−^/PARP2^−/−^* RPE-1 cells, in which SSBR rates were consistently reduced throughout the experiment (Figure 2B). We did not observe an accumulation of DNA breaks following CPT treatment, which induces SSBs resulting from abortive topoisomerase 1 (TOP1) activity, in any HPF1-deficient cells (Supplementary Figure S2C). However, *HPF1^−/−^* cells accumulated higher levels of DNA strand breaks after MMS treatment, an alkylating agent where SSBs arise as intermediates of DNA base excision repair (BER) (Figure 2C and D). Despite this, the repair of these breaks was not affected in HPF1-deficient cells, in contrast to the pronounced defects observed in *PARP1^−/−^/PARP2^−/−^* cells. In summary, our experiments lead us to conclude that HPF1 is largely dispensable for the repair of various physiologically relevant SSBs. While the absence of HPF1 may impact the accumulation of SSB intermediates following specific genotoxins in particular cell lines at the initial stages, overall SSBR is not dependent on HPF1.

### Robust poly-ADP-ribosylation during SSBR in HPF1-deficient cells

**Figure 3.**
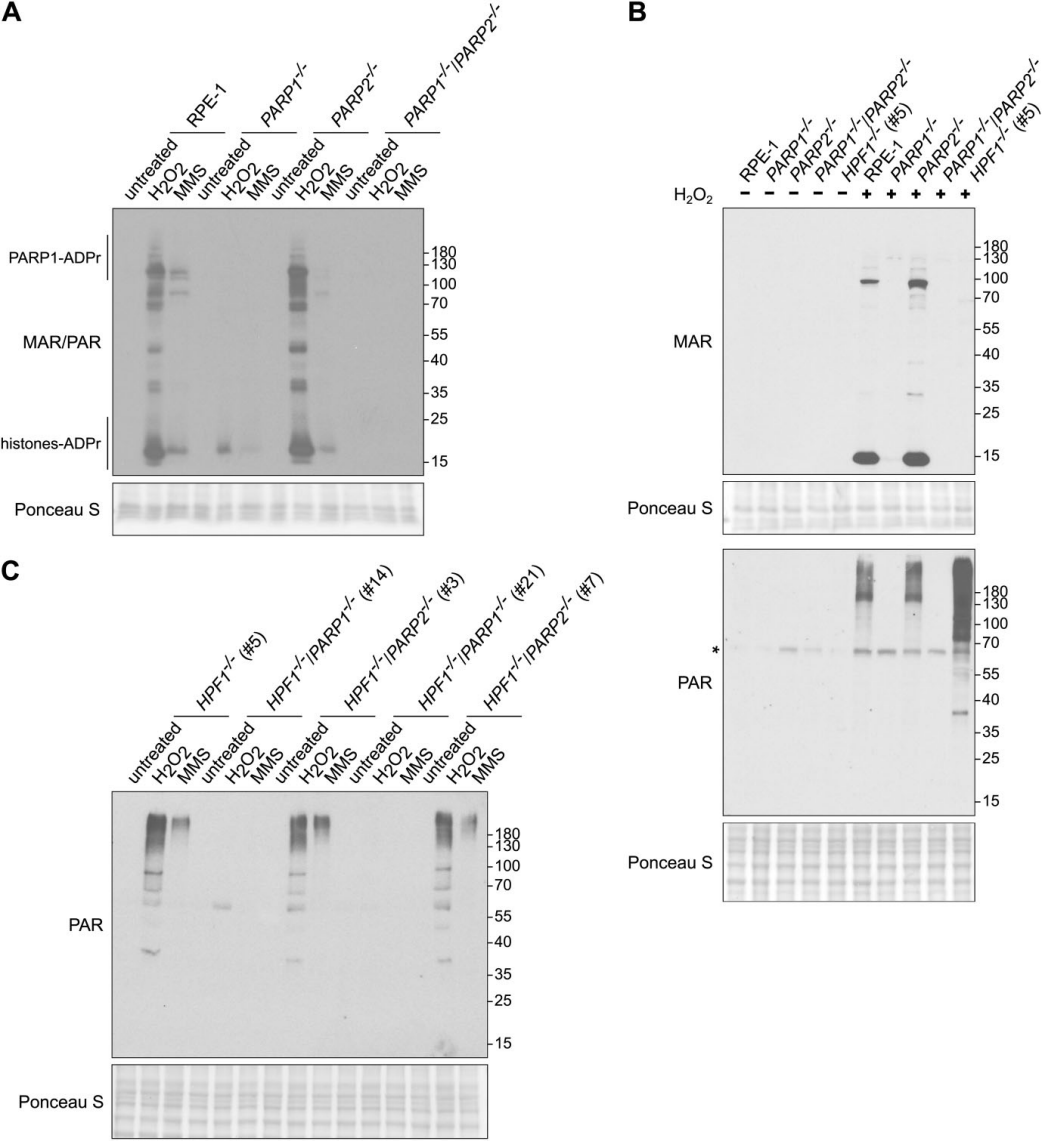
PARP1-dependent ADP-ribosylation following DNA damage in both wild-type and HPF1-deficient cells. (**A**) ADP-ribosylation levels in RPE-1 wild-type, *PARP1^−/−^* (clone #G7), *PARP2^−/−^* (clone #A1), and *PARP1^−/−^/PARP2^−/−^* (clone #E6) cells after treatment with 2 mM H_2_O_2_ for 20 min or 0.9 mM MMS for 1 hour in full media at 37°C detected by western blotting using the iAf1521 reagent (MAR/PAR). (**B**) ADP-ribosylation levels in RPE-1 wild-type, *PARP1^−/−^* (clone #G7), *PARP2^−/−^* (clone #A1), *PARP1^−/−^/PARP2^−/−^* (clone #E6) and *HPF1^−/−^* (clone #5) cells before and after treatment with 2 mM H_2_O_2_ for 20 min in full media at 37°C detected by western blotting using a specific anti-mono-ADP-ribose binding reagent (MAR 647) or anti-poly-ADP-ribose antibody (PAR). The *asterisk* denotes a nonspecific band, resulting from a cross-reaction with a component from the serum. (**C**) ADP-ribosylation levels in *HPF1^−/−^* (clone #5), *HPF1^−/−^/PARP1^−/−^* (clone #14 and #21), and *HPF1^−/−^/PARP2^−/−^* (clone #3 and #7) cells after treatment with 2 mM H_2_O_2_ for 20 min or 0.9 mM MMS for 1 hour in full media at 37°C detected by western blotting using a specific anti-poly-ADP-ribose antibody (PAR).

**Figure 4.**
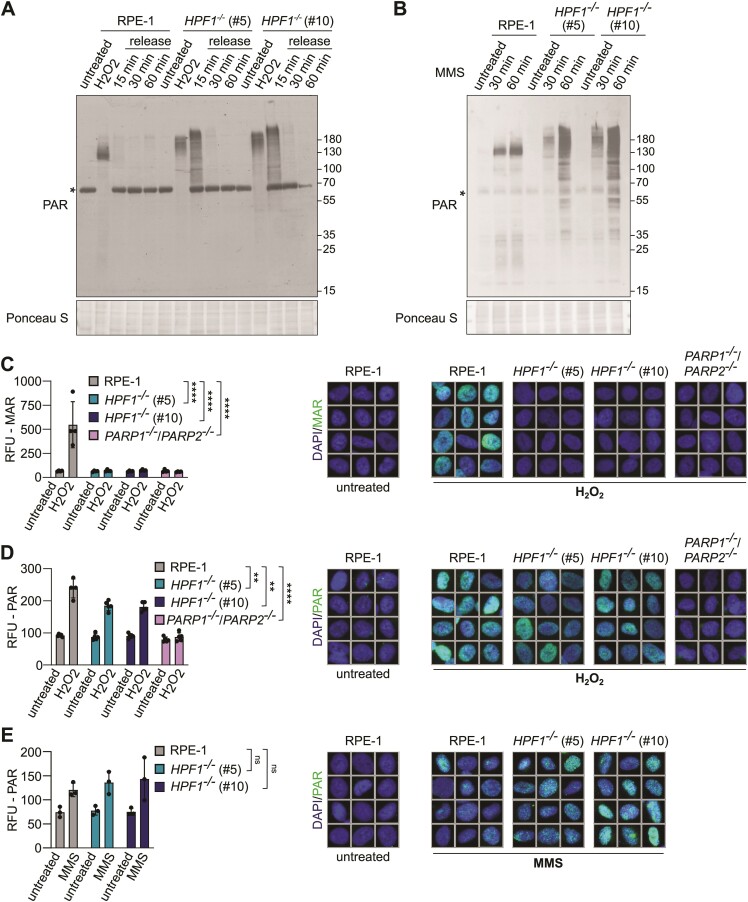
The elongated poly-ADP-ribose chains in HPF1-deficient cells after DNA damage. (**A**) Poly-ADP-ribosylation in RPE-1 wild-type and *HPF1^−/−^* (clone #5 and #10) cells after 100 μM H_2_O_2_ treatment in serum-free media for 10 min on ice, followed by incubation at 37°C in full media (release time) detected by western blotting using a specific anti-poly-ADP-ribose antibody (PAR). The *asterisk* denotes a nonspecific band, resulting from a cross-reaction with a component from the serum. (**B**) Poly-ADP-ribosylation in RPE-1 wild-type and *HPF1^−/−^* (clone #5 and #10) cells after treatment with 0.9 mM MMS for 30 and 60 min at 37°C detected by western blotting using a specific anti-poly-ADP-ribose antibody (PAR). The *asterisk* denotes a nonspecific band, resulting from a cross-reaction with a component from the serum. (**C**, **D**) Immunofluorescence analysis of ADP-ribosylation levels detected by the specific mono-ADP-ribosylation (MAR 205) binding reagent or the specific poly-ADP-ribosylation (PAR) antibody after detergent-extraction in untreated RPE-1 wild-type, *HPF1^−/−^* (clone #5 and #10), and *PARP1^−/−^/PARP2^−/−^* (clone #E6) cells and after treatment with 2 mM H_2_O_2_ in full media for 20 min at 37°C. Data represents the mean (±SD) of four independent experiments, RFU—relative fluorescence units. Statistical analysis (one-way analysis of variance) is shown (**P < 0.01, ****P < 0.0001). The corresponding representative ScanR images for immunofluorescence data are shown. (**E**) Immunofluorescence analysis of ADP-ribosylation levels detected by the specific poly-ADP-ribosylation (PAR) antibody after detergent-extraction in untreated RPE-1 wild-type and *HPF1^−/−^* (clone #5 and #10) cells and after treatment with 0.9 mM MMS for 1 h at 37°C. Data represents the mean (±SD) of three independent experiments, RFU – relative fluorescence units. Statistical analysis (one-way analysis of variance) is shown (ns, not significant). The corresponding representative ScanR images for immunofluorescence data are shown.

Given the established importance of PARP1 and/or PARP2 for SSBR (13,37), we aimed to understand the relative dispensability of HPF1 for this process, and thus compared the levels of ADP-ribosylation in cells following DNA damage. In order to distinguish the specific contributions of PARP1 and PARP2 to ADP-ribosylation in wild-type and HPF1-deficient cells, we employed a panel of CRISPR/Cas9-edited RPE-1 cell lines (Supplementary Figure S1A). As previously suggested, the majority of ADP-ribosylation induced by H_2_O_2_ or MMS was dependent on PARP1 (Figure 3A, Supplementary Figure S3A). Additionally, using mono-specific and poly-specific ADP-ribose binding reagents, we observed PARP1-dependency for both mono-ADP-ribosylation (MAR) and poly-ADP-ribosylation (PAR) following treatment with both genotoxins (Figure 3B, Supplementary Figure S3B and C). Similarly, when investigating ADP-ribosylation induced by DNA damaging agents in HPF1-deficient cells, we found that it was primarily dependent on PARP1 (Figure 3C). Importantly, we noticed that whereas MAR levels were greatly reduced in HPF1-deficient cells, there was an increase in PAR levels (Figure 3B). As expected, we did not detect ADP-ribosylation of histones in H_2_O_2_ and MMS-treated *HPF1^−/−^* cells. On the contrary, we observed high levels of auto-poly-ADP-ribosylated PARP1 (Figure 4A and B; Supplementary Figure S4A and B). Interestingly, the length and/or complexity of the PAR chains on auto-poly-ADP-ribosylated PARP1 appeared to increase in HPF1-deficient cells, as indicated by slower electrophoretic mobility compared to wild-type cells. Particularly noteworthy is the pronounced increase observed in HPF1-deficient RPE-1 cells 15 min after H_2_O_2_ treatment (Figure 4A). This observation aligns well with the slight delay in SSBR at this time point, suggesting that the elongated poly-ADP-ribose chains may partially impede the repair process. Consistent with previously published data (25), this residual poly-ADP-ribosylation in *HPF1^−/−^* cells was primarily localized on non-serine residues, as indicated by its sensitivity to hydroxylamine (Supplementary Figure S4C).

Subsequently, we investigated the levels of chromatin-associated mono- and poly-ADP-ribosylation by immunofluorescence in both control and HPF1-deficient cells after DNA damage to assess the potential for recruitment of DNA repair factors to SSBs. These experiments revealed a significant increase in the levels of chromatin-bound MAR and PAR in wild-type U2OS and RPE-1 cells following H_2_O_2_ treatment, and as expected, these levels were abolished in PARP1/PARP2-deficient cells (Figure 4C and D; Supplementary Figure S4D and E). Importantly, in the HPF1-deficient U2OS and RPE-1 cells, MAR levels were significantly reduced upon H_2_O_2_ treatment, while the decrease in PAR levels was relatively modest. Similarly, the levels of MMS-induced chromatin-bound PAR were not greatly affected by HPF1 deletion (Figure 4E; Supplementary Figure S4F). In summary, our data suggest that residual poly-ADP-ribosylation at the chromatin of HPF1-deficient cells following DNA damage may be sufficient for the recruitment of DNA repair factors, thereby facilitating efficient DNA repair.

### XRCC1 accumulation to chromatin of HPF1-deficient cells remains unaffected

**Figure 5.**
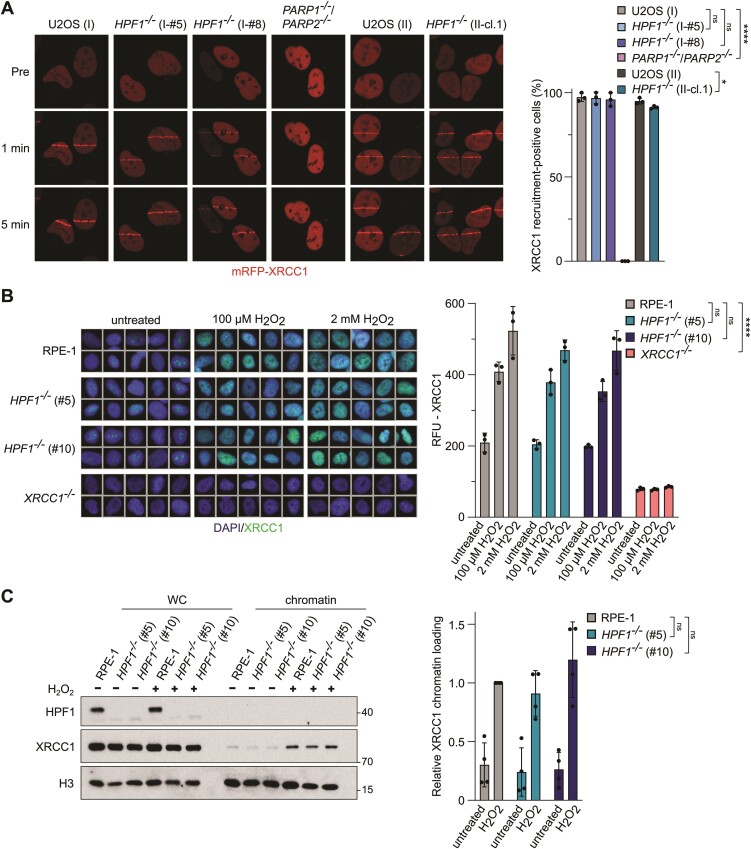
XRCC1 recruitment into chromatin following DNA damage remains unaffected in HPF1-deficient cells. (**A**) U2OS (I) wild-type, *HPF1^−/−^* (clone I-#5 and I-#8), *PARP1^−/−^/PARP2^−/−^* (clone #5), U2OS (II) wild-type, and *HPF1^−/−^* (II-cl.1) cells were transiently transfected with mRFP-XRCC1 and microirradiated with a 405 nm UV-laser. Representative images captured pre-irradiation, and at one and five minutes post-irradiation, and quantifications are shown. Data represents the mean percentage (±SD) of transfected cells with XRCC1 recruitment to DNA damage sites, averaged over three independent experiments. Statistical analysis (one-way analysis of variance) is shown (ns, not significant, *P < 0.05, ****P < 0.0001). A minimum of 17 transfected cells were analysed per sample in each experiment. (**B**) Immunofluorescence analysis of chromatin-bound nuclear XRCC1 after detergent-extraction in RPE-1 wild-type, *HPF1^−/−^* (clone #5 and #10), and *XRCC1^−/−^* (clone #3) cells untreated or treated with indicated doses of H_2_O_2_ in serum-free media for 10 min on ice. Representative ScanR images and quantifications are shown, RFU—relative fluorescence units. Data are the mean (±SD) of three independent experiments. Statistical analysis (one-way analysis of variance) is shown (ns, not significant, ****P < 0.0001). (**C**) Levels of HPF1 and XRCC1 in whole cell (WC) extracts and chromatin-containing fractionations from RPE-1 wild-type, *HPF1^−/−^* (clone #5 and #10) cells before and following treatment with 2 mM H_2_O_2_ in full media for 20 min at 37°C. Normalized chromatin bound XRCC1 protein levels, quantified from the western blots, represent the mean (±SD) from four independent experiments. Statistical analysis (one-way analysis of variance) is shown (ns, not significant).

One critical function of PARP activity in response to SSBs induced by genotoxic stress is to facilitate the rapid recruitment of XRCC1 protein complexes, ultimately promoting efficient SSBR (13,38). To assess whether the remaining non-serine poly-ADP-ribosylation in HPF1-deficient cells is sufficient to recruit XRCC1 to sites of DNA damage, we conducted laser-microirradiation experiments in cells transfected with XRCC1 fused to monomeric RFP (red fluorescent protein) to monitor recruitment in living cells. Interestingly, XRCC1 recruitment was intact in U2OS and RPE-1 cell lines, including wild-type and HPF1-deficient cells, except for PARP1/PARP2-deficient cells, where XRCC1 recruitment was completely blocked, as expected (Figure 5A; Supplementary Figure S5A).

To confirm that this phenomenon extends to endogenous XRCC1 in HPF1-deficient cells, we conducted immunofluorescent analyses of chromatin-bound XRCC1 following SSB induction in non-transfected cells. Remarkably, consistent with the normal rate of SSBR observed in *HPF1^−/−^* U2OS cells, we found that the PARP-dependent accumulation of XRCC1 in chromatin following H_2_O_2_ treatment remained unaffected in *HPF1^−/−^* U2OS cells (Supplementary Figure S5B). Moreover, the recruitment of XRCC1 to DNA damage sites was similarly unaffected in HPF1-deficient RPE-1 cells (Figure 5B). These findings were further supported when we examined XRCC1 recruitment to oxidized chromatin using biochemical fractionation (Figure 5C; Supplementary Figure S5C). Collectively, our findings suggest that HPF1 is largely dispensable for the repair of SSBs in response to oxidative stress, as evidenced by the unaffected recruitment of XRCC1 to DNA damage sites in HPF1-deficient cells.

In summary, the loss of HPF1 did not consistently increase cellular sensitivity to various genotoxins, despite the requirement for PARP1 activity. Although serine mono-ADP-ribosylation was reduced, HPF1-deficient cells retained nearly normal levels of chromatin poly-ADP-ribosylation following SSBs induction, allowing the recruitment of the DNA repair factor XRCC1. Thus, the rate of SSBR in HPF1-deficient cells was largely unaffected after exposure to genotoxins, with only a mild effect on the induction and/or early repair of SSB intermediates, depending on the cell line. This mild defect might be due to an indirect impact of altered PAR chain length in HPF1-deficient cells on SSBR, potentially by altering the dynamics of chromatin relaxation. Overall, these data suggest that HPF1 and histone serine ADP-ribosylation are largely dispensable for the direct PARP1-dependent SSBR in human cells.

## Discussion

The recent discovery of histone PARylation factor-1 (HPF1) has revealed a potentially new important component of the activities of PARP1 and PARP2 in the context of DNA damage response (21). Upon DNA damage, HPF1 associates with PARP1/2 at the DNA break sites, profoundly influencing their behaviour (24). This interaction redirects their substrate specificity from various amino acids to a preference for serine residues, highlighting the possible importance of HPF1-dependent serine ADP-ribosylation during DNA repair (22). However, the direct impact of HPF1 deficiency on DNA repair efficiency, particularly in single-strand break repair (SSBR), has remained unexplored until now.

Surprisingly, contrary to our initial expectations based on previous reports (21,36), our findings suggest that HPF1-mediated serine ADP-ribosylation is relatively dispensable for the role/s of PARP1/PARP2 in maintaining cellular resistance to various genotoxins. We revealed that the absence of HPF1 did not generally increase cellular sensitivity to hydrogen peroxide (H_2_O_2_), camptothecin (CPT), or methyl methanesulfonate (MMS), that collectively induce a range of DNA single-strand breaks (SSBs) requiring PARP1/2 activity for their repair. Importantly, unlike HPF1-deficient cells, those lacking both PARP1 and HPF1, or PARP1/2 showed hypersensitivity to SSB-inducing genotoxins, implying that HPF1 deficiency may not substantially impact PARP-dependent DNA repair following the induction of SSBs. Consistent with this conclusion, measurements for SSBR rates using alkaline comet assays failed to detect robust defects in HPF1-deficient cell lines following treatment with H_2_O_2_, MMS or CPT. We observed a slower DNA repair only at early time point after H_2_O_2_ exposure in *HPF1^−/−^* RPE-1 cells. This defect was absent from *HPF1^−/−^* U2OS cells, and was considerably milder than the impairment seen in PARP1/PARP2-deficient cells. While SSBR was not generally affected following treatment with the alkylating agent MMS in HPF1-deficient cells compared to PARP1/2-deficient cells, alkaline comet assays revealed an increase in the accumulation of SSBs in both HPF1-deficient U2OS and RPE-1 cells after the treatment. The observed alterations in poly-ADP-ribosylation dynamics in HPF1-deficient cells could contribute to a transient and slight delay in DNA repair during the initial stages, leading to accumulation of SSBs during continuous MMS treatment, until the ratio of poly-ADP-ribose is properly balanced to facilitate the appropriate recruitment of DNA repair factors near the DNA damage sites. This aligns with *in vitro* data where HPF1 not only facilitates ADP-ribose targeting to specific residues but also modulates the rate of polymerization, favouring mono-ADP-ribose modifications over poly-ADP-ribose chains (39).

We detected no significant differences in the loading of the downstream single-strand break repair factor XRCC1 to chromatin following few minutes after DNA damage. These data strongly imply that HPF1 is dispensable for the PARP-dependent recruitment of the core SSBR proteins and its associated factors, and certainly for cellular sensitivity to SSBs. As expected, the deletion of HPF1 ablated histone ADP-ribosylation (24). However, in contrast, the auto-poly-ADP-ribosylation of PARP1, presumably on aspartate/glutamate and/or other non-serine residues, remained robust and/or slightly increased. This suggests that serine ADP-ribosylation, and histone ADP-ribosylation in general, is dispensable for the recruitment of XRCC1 protein complexes and that PARP1 auto-poly-ADP-ribosylation is sufficient for XRCC1 recruitment and SSBR following exposure to SSB-inducing genotoxins.

In summary, our study suggests that HPF1, and consequently serine ADP-ribosylation, and histone ADP-ribosylation are largely dispensable for PARP1-dependent SSBR in human cells and for cellular resistance to various SSB inducing agents. We propose that, following DNA damage, although serine ADP-ribosylation occurs rapidly and abundantly after PARP1 activation by SSB induction in the presence of HPF1, it is the auto-poly-ADP-ribosylation of non-serine residues that plays a crucial role in DNA repair by swiftly recruiting DNA single-strand break repair factors. Alternatively, it is possible that the recruitment of SSBR factors does not discriminate between ADP-ribosylated serine and other residues. If these hypotheses hold true, the question arises: what is the cellular purpose of HPF1? We posit that serine ADP-ribosylation, particularly on histones, mediated by HPF1, likely fulfils other PARP-dependent functions before and/or after DNA damage, such as the chromatin remodelling and regulation of chromatin compaction during transcriptional regulation (18,36,40–42).

These findings significantly contribute to our comprehension of the intricate mechanisms governing DNA repair. They highlight the compensatory mechanisms that come into play when specific components, such as HPF1-mediated serine ADP-ribosylation, are disrupted. Understanding the importance of serine-ADP-ribosylation is crucial, given its potential toxicity in the brain of patients with mutations in ARH3, the enzyme responsible for its removal (18,43). Under normal conditions, the absence of active ARH3 doesn’t impact SSBR, however, it leads to the accumulation of potentially harmful mono-ADP-ribose on histones near DNA repair sites as remnants of PARP activity, signifying inherent DNA damage. These alterations might disrupt the established histone code, potentially interfering with transcription and triggering cellular dysfunction. Alternatively, they might serve as a platform, initiating a poly-ADP-ribose chain reaction that depletes NAD+, possibly contributing to neurological complications (18,44). Further exploration into the broader implications of these mechanisms holds the promise of deeper insights into cellular functions, particularly in complex systems like the brain.

## Supplementary Material

gkae708_Supplemental_File

## Data Availability

The data underlying this study is available in the article and its online supplementary material.
