# Supplementary material for: Dispensability of HPF1 for cellular removal of DNA single-strand breaks
Source: Nucleic Acids Res. 2024 Aug 20;52(18):10986–98. doi: 10.1093/nar/gkae708 (PMC11472159; doi:10.1093/nar/gkae708)
Supplement: gkae708_Supplemental_File [file gkae708_supplemental_file.pdf]

Supplementary Data

Dispensability of HPF1 for Cellular Removal of DNA Single-Strand Breaks

Hrychova & Burdova et al.

**Supplementary Table S1.**	CRISPR/Cas9 gene-edited cell lines employed
**Supplementary Table S2.**	RNA guides sequences for CRISPR/Cas9 cloning
**Supplementary Table S3.**	Primary and secondary antibodies for immunofluorescence and Western blotting

**Supplementary Figure S1.**	The accumulation of endogenous mono-ADP-ribose in ARH3-deficient cells
**Supplementary Figure S2.**	Single-strand break repair in HPF1-deficient cells (Related to Figure 1 and 2)
**Supplementary Figure S3.**	PARP1-dependent ADP-ribosylation following DNA damage (Related to Figure 3)
**Supplementary Figure S4.**	DNA damage induced poly-ADP-ribosylation in HPF1-deficient cells (Related to Figure 4)
**Supplementary Figure S5.**	XRCC1 recruitment into chromatin following DNA damage in HPF1-deficient cells (Related to Figure 5)

Table S1. CRISPR/Cas9 gene-edited cell lines employed

**Cell line**	clone	source
hTERT RPE-1 *PARP1*$^{-/-}$	#G7	Hanzlikova et al. 2017
hTERT RPE-1 *PARP2*$^{-/-}$	#A1	Hanzlikova et al. 2017
hTERT RPE-1 *PARP1*$^{-/-}$/*PARP2*$^{-/-}$	#E6	Hanzlikova et al. 2017
hTERT RPE-1 *XRCC1*$^{-/-}$	#3	Hoch et al. 2017
hTERT RPE-1 *HPF1*$^{-/-}$	#5	This study
hTERT RPE-1 *HPF1*$^{-/-}$	#10	This study
hTERT RPE-1 *HPF1*$^{-/-}$/*PARP1*$^{-/-}$	#14	This study
hTERT RPE-1 *HPF1*$^{-/-}$/*PARP1*$^{-/-}$	#21	This study
hTERT RPE-1 *HPF1*$^{-/-}$/*PARP2*$^{-/-}$	#3	This study
hTERT RPE-1 *HPF1*$^{-/-}$/*PARP2*$^{-/-}$	#7	This study
hTERT RPE-1 *ARH3*$^{-/-}$	#1	This study
U2OS *HPF1*$^{-/-}$	cl.1	Gibbs-Seymour et al. 2016
U2OS *HPF1*$^{-/-}$	#5	This study
U2OS *HPF1*$^{-/-}$	#8	This study
U2OS *ARH3*$^{-/-}$	#48	Fontana et al. 2017
U2OS *HPF1*$^{-/-}$/*ARH3*$^{-/-}$	#D	This study
U2OS *PARP1*$^{-/-}$	#15	This study
U2OS *PARP2*$^{-/-}$	#5	This study
U2OS *PARP1*$^{-/-}$/*PARP2*$^{-/-}$	#5	This study
U2OS *XRCC1*$^{-/-}$	#2	Polo et al. 2019

Table S2. RNA guides sequences for CRISPR/Cas9 cloning

**gRNA**	sequence	type	source
HPF1#1	5´-tcggcggtggcgggaagcgc-3´	plasmid DNA	Ahel Lab
HPF1#2	5´-cagcagaattccccgatccg-3´	plasmid DNA	Ahel Lab
ARH3#1	5'-GCAGCCTCGGAAGCGCGAGA-3'	plasmid DNA	Ahel Lab
ARH3#2	5'-GGGCGAGACGTCTATAAGGC-3'	plasmid DNA	Ahel Lab
PARP1	5'-CGAGUCGAGUACGCCAAGAG-3'	synthetic RNA	
PARP2#1	5'-ACGUCAGCGUUCGAAUUCCA-3'	synthetic RNA	
PARP2#2	5'-GGAGUCGAAAAAGAUGCCUG-3'	synthetic RNA	

Table S3. Primary and secondary antibodies for immunofluorescence and Western blotting

**Primary antibodies**	**Source**	**Identifier**
MAR (#204)	Biorad	HCA354
MAR (#205)	Biorad	HCA355
MAR (#647)	Biorad	TZA020
PAR	Trevigen	4336-BPC-100
MAR/PAR – iAf1521	This study	
HPF1	Novus Biologicals	NBP1-93973
ARH3	Sigma-Aldrich	HPA027104
PARP1	Serotec	MCA1522G
PARP2	Active Motif	39743
XRCC1	Novus Biologicals	NBP1-87154
Histone 3 (H3)	Abcam	ab10799
Tubulin	Abcam	ab6160
Importin β	Santa Cruz	sc-137016
**Secondary antibodies**	**Source**	**Identifier**
Goat anti-mouse HRP	Biorad	170-6516
Goat anti-rabbit HRP	Biorad	170-6516
Donkey anti-mouse AlexaFluor 647	Life Technologies	A31571
Donkey anti-rabbit AlexaFluor 488	Life Technologies	A21206

Supplementary Figure S1

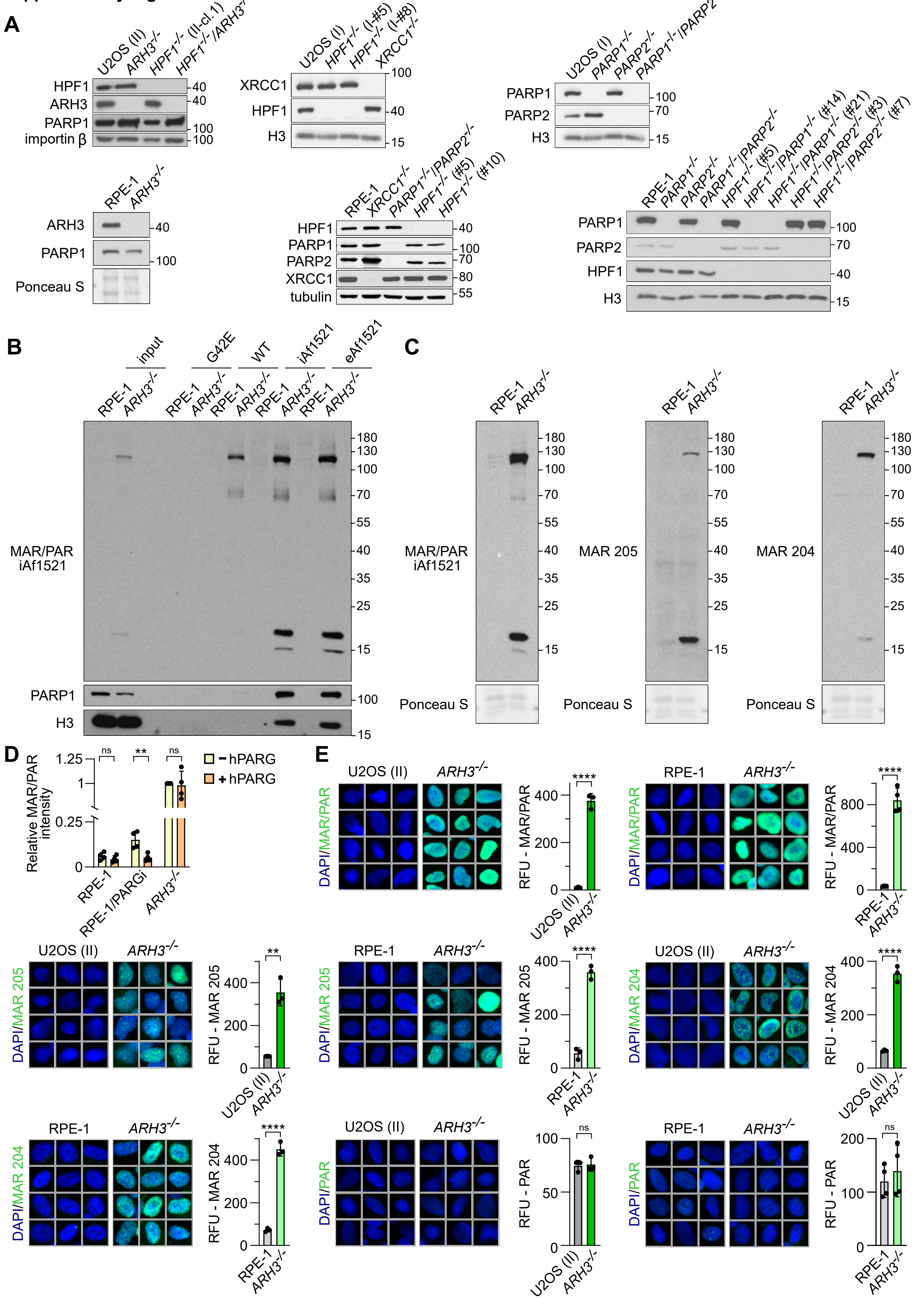

Supplementary figure S2

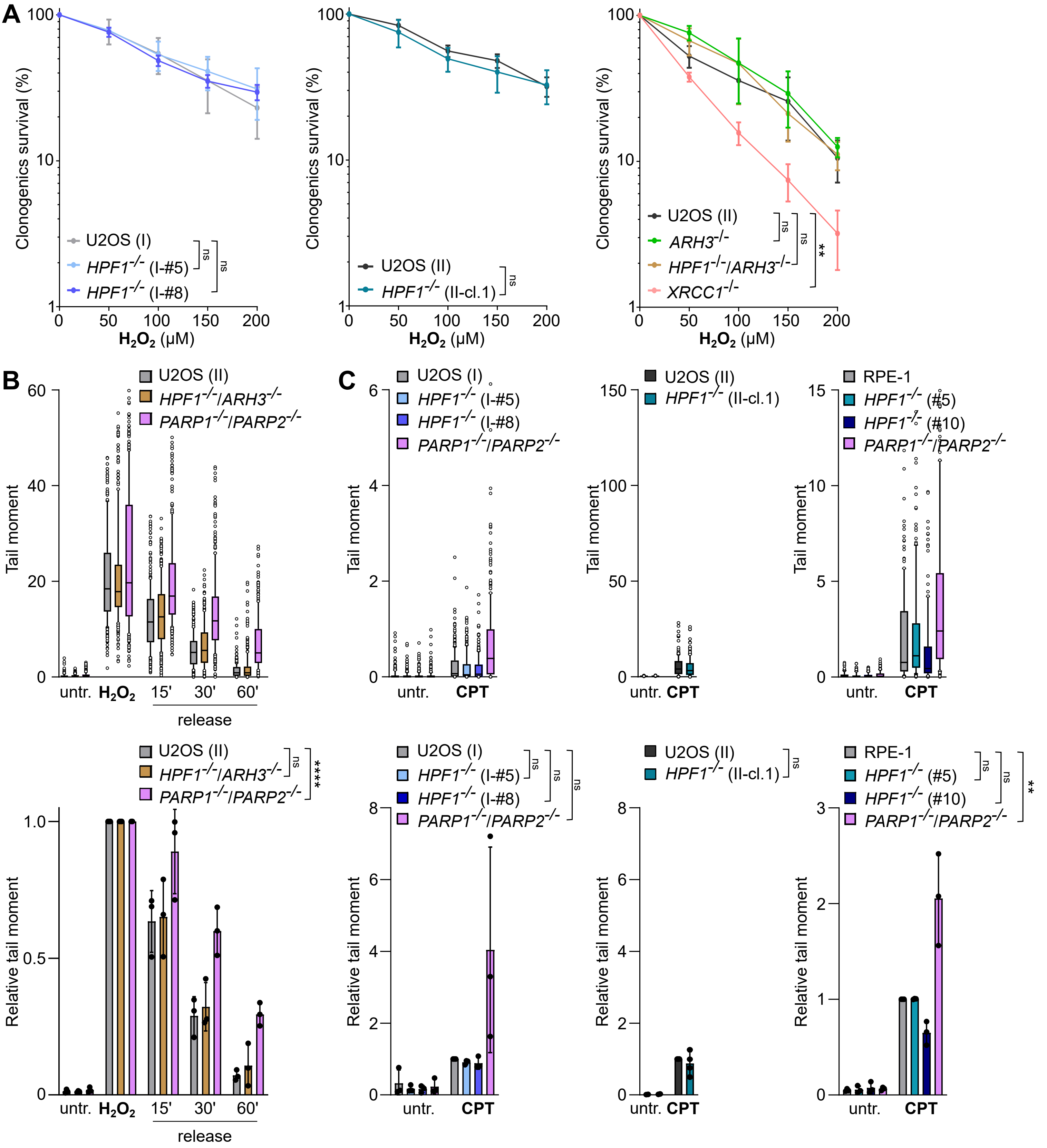

Supplementary Figure S3

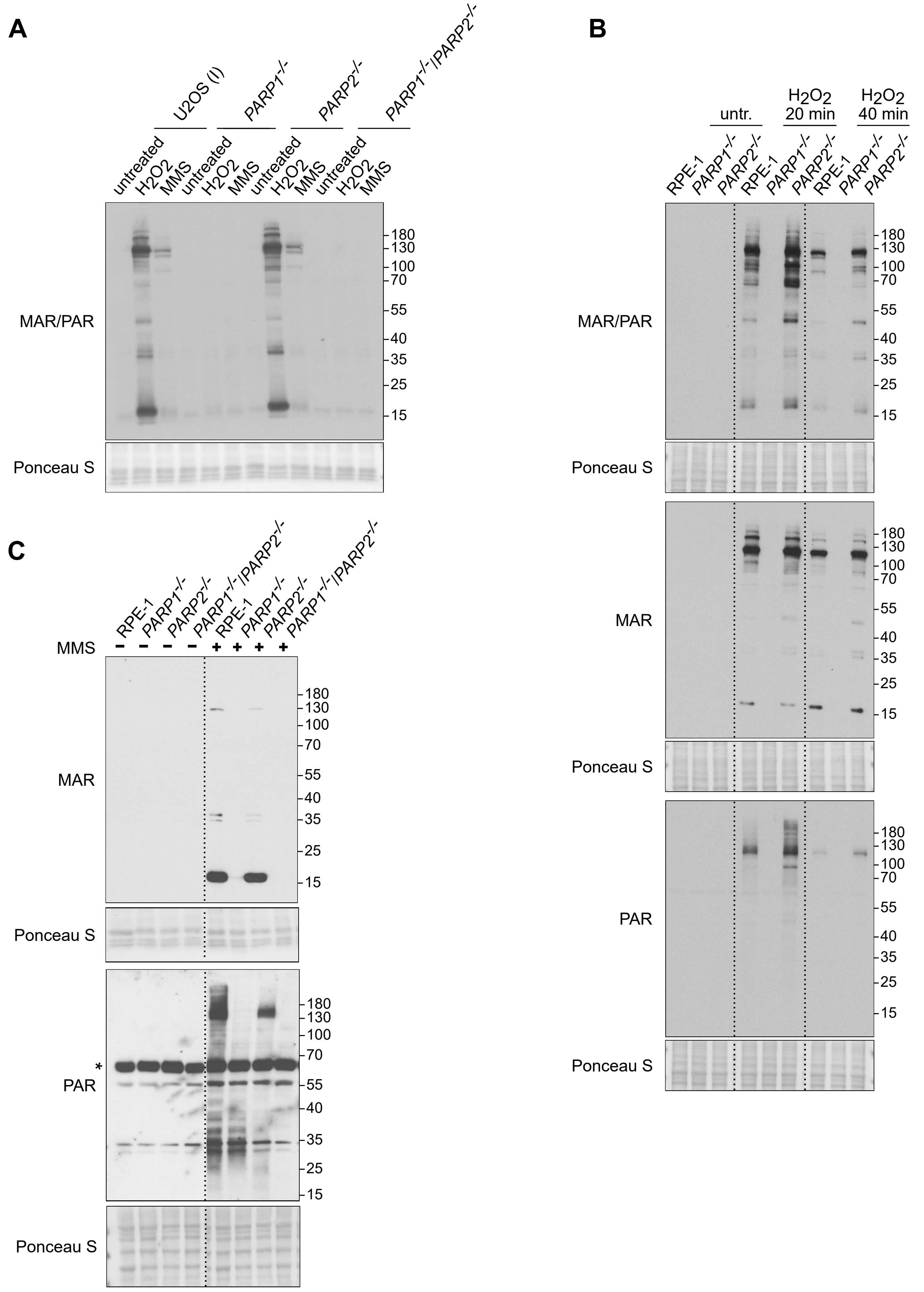

Supplementary Figure S4

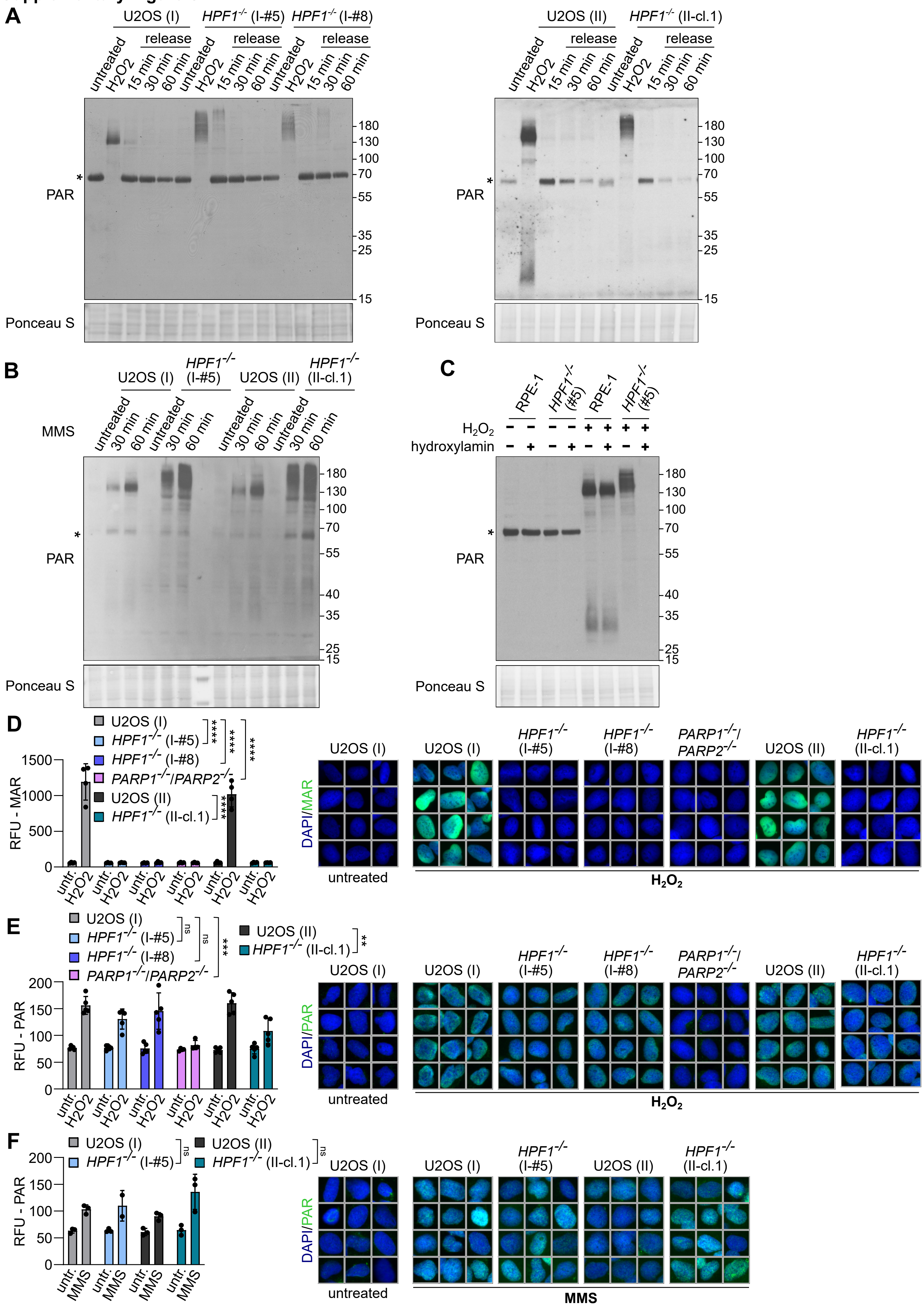

Supplementary Figure S5

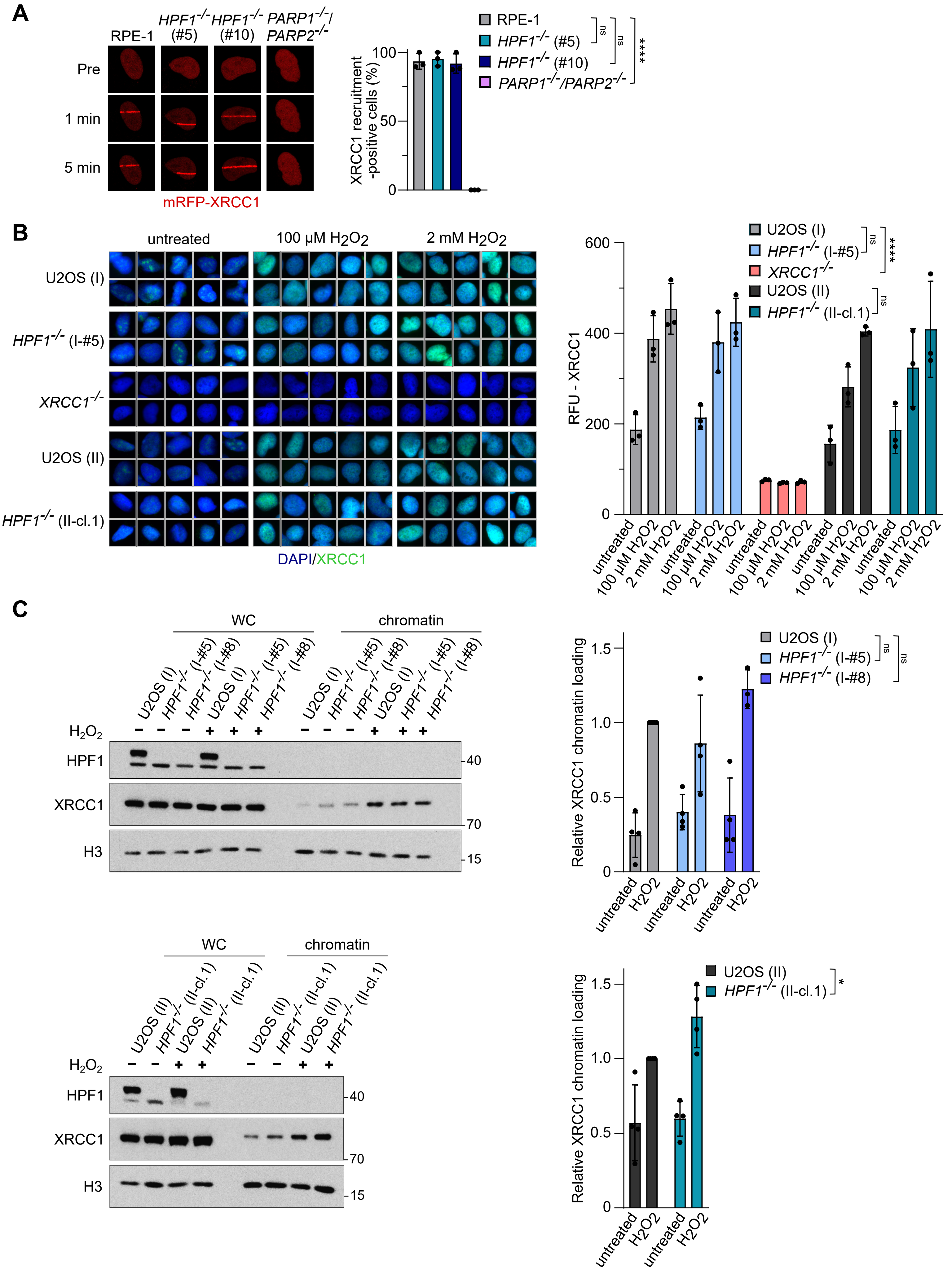

Supplementary Figure S1. The accumulation of endogenous mono-ADP-ribose in ARH3-deficient cells. (**A**) Western blot analysis of the indicated proteins in indicated U2OS and RPE-1 CRISPR/Cas9 gene edited cell lines. (**B**) ADP-ribosylation levels detected by western blotting using the iAf1521 reagent (MAR/PAR) in wild-type RPE-1 and *ARH3*$^{-/-}$ (clone #1) cell lysates. Samples were analysed before (input) and after immunoprecipitation with beads coupled with the indicated Af1521 variant (mutated G24E, wild-type WT, iAf1521, and eAf1521). (**C**) Detection of endogenous ADP-ribose levels in wild-type RPE-1 and *ARH3*$^{-/-}$ (clone #1) cells by western blotting using the iAf1521 reagent (MAR/PAR) and specific mono-ADP-ribose 205 (MAR 205) or mono-ADP-ribose 204 (MAR 204) binding reagents, as indicated. (**D**) Immunofluorescence analysis of ADP-ribosylation levels detected by the iAf1521 reagent (MAR/PAR) after detergent-extraction in RPE-1 wild-type cells, RPE-1 cells treated with a 10 μM PARG inhibitor (PARGi) for 30 min, and *ARH3*$^{-/-}$ (clone #1) cells. Following fixation, samples were either left untreated (-) or treated (+) with 200 nM human purified PARG protein (hPARG) for 1 hour at 37 °C. Relative quantifications are shown, representing the mean (±SD) of four independent experiments. Statistical significance was determined by two-tailed unpaired Student's t-test (ns - not significant, $^{**}P < 0.01$). (**E**) Immunofluorescence analysis of endogenous ADP-ribosylation levels detected by the iAf1521 reagent (MAR/PAR), specific mono-ADP-ribose 205 (MAR 205) or mono-ADP-ribose 204 (MAR 204) binding reagents, and poly-ADP-ribose (PAR) antibodies after detergent-extraction in U2OS (II) wild-type, *ARH3*$^{-/-}$ (clone #48) and RPE-1 wild-type, *ARH3*$^{-/-}$ (clone #1) cells, as indicated. Representative ScanR images and quantifications are shown, RFU - relative fluorescence units. Data are the mean (±SD) of three to four independent experiments. Statistical significance was determined by two-tailed unpaired Student's t-test (ns - not significant, $^{**}P < 0.01$, $^{****}P < 0.0001$).

Supplementary Figure S2. Single-strand break repair in HPF1-deficient cells (Related to Figure 1 and 2). (**A**) Clonogenic survival assay in U2OS (I) wild-type, *HPF1*$^{-/-}$ (clone I-#5 and I-#8), and U2OS (II) wild-type, *HPF1*$^{-/-}$ (II-cl.1), and *ARH3*$^{-/-}$ (clone #48), *ARH3*$^{-/-}$/*HPF1*$^{-/-}$ (clone #D), and *XRCC1*$^{-/-}$ (clone #2) cells, as indicated, in response to treatment with indicated doses of H_2O_2 in serum-free media for 10 min at RT. Data represent the mean (±SD) of three to six independent experiments. Statistical analysis (two-way analysis of variance) is shown (ns – not significant, $^{**}P < 0.01$). (**B**) Alkaline comet assay analysis (*Methods I*) depicting the repair of DNA single-strand breaks (SSBs) induced by 100 μM H_2O_2 treatment in serum-free media for 10 min on ice, followed by incubation at 37 °C in full media in U2OS (II) wild-type, *ARH3*$^{-/-}$*HPF1*$^{-/-}$ (clone #D), and *PARP1*$^{-/-}$*PARP2*$^{-/-}$ (clone #5) cells. The individual comet tail moments of cells combined from three independent experiments are plotted (*upper chart*). A minimum of 50 cells were analysed per sample in each of the experiments. The normalized data are shown (*the lower chart*) and represent the relative mean (±SD) of three independent experiments. Statistical analysis (two-way analysis of variance) is shown (ns – not significant, $^{****}P < 0.0001$). (**C**) Alkaline comet assay analysis (*Methods I)* of SSBs induced by treatment with 10 μM CPT for 45 min at 37 °C in U2OS or RPE-1 clones, as indicated. The individual comet tail moments of cells combined from three to four independent experiments are plotted (*the upper chart*). A minimum of 50 cells were analysed per sample in each of the experiments. The normalized data are shown (*the lower chart*) and represent the relative mean (±SD) of three to four independent experiments. Statistical analysis (one-way analysis of variance) is shown (ns – not significant, $^{**}P < 0.01$).

Supplementary Figure S3. PARP1-dependent ADP-ribosylation following DNA damage (Related to Figure 3). (**A**) ADP-ribosylation levels in U2OS wild-type (I), *PARP1*$^{-/-}$ (clone #15), *PARP2*$^{-/-}$ (clone #5), and *PARP1*$^{-/-}$*PARP2*$^{-/-}$ (clone #5) cells after treatment with 2 mM H_2O_2 at 37 °C in full media for 20 min and 0.9 mM MMS at 37 °C for 1 hour detected by western blotting using the iAf1521 reagent (MAR/PAR). (**B**) ADP-ribosylation levels in RPE-1 wild-type, *PARP1*$^{-/-}$ (clone #G7), and *PARP2*$^{-/-}$ (clone #A1) cells after treatment with 200 μM H_2O_2 at 37 °C in full media for 20 min or 40 min detected by western blotting using the iAf1521 reagent (MAR/PAR) or mixture of specific anti-mono-ADP-ribose binding reagents (MAR, 204 and 205) or anti-poly-ADP-ribose antibody (PAR). (**C**) Mono- and poly-ADP ribosylation in RPE-1 wild-type, *PARP1*$^{-/-}$ (clone #G7), *PARP2*$^{-/-}$ (clone #A1), and *PARP1*$^{-/-}$*PARP2*$^{-/-}$ (clone #E6) cells after treatment with 0.9 mM MMS at 37 °C for 1 hour detected by western blotting using a specific anti-mono-ADP-ribose binding reagent (MAR 647) or anti-poly-ADP-ribose antibody (PAR). The *asterisk* denotes a nonspecific band, resulting from a cross-reaction with a component from the serum.

Supplementary Figure S4. DNA damage induced poly-ADP-ribosylation in HPF1-deficient cells (Related to Figure 4). (**A**) Poly-ADP-ribosylation levels in U2OS (I) wild-type and *HPF1*$^{-/-}$ (clone I-#5 and I-#8), and U2OS (II) wild-type, and *HPF1*$^{-/-}$ (II-cl.1) cells after 100 µM H_2O_2 treatment in serum-free media for 10 min on ice, followed by incubation at 37 °C in full media (release time) detected by western blotting using a specific anti-poly-ADP-ribose antibody (PAR). The *asterisk* denotes a nonspecific band, resulting from a cross-reaction with a component from the serum. (**B**) Poly-ADP-ribosylation levels in U2OS (I) wild-type and *HPF1*$^{-/-}$ (clone I-#5), U2OS (II) wild-type, and *HPF1*$^{-/-}$ (II-cl.1) cells after treatment with 0.9 mM MMS at 37 °C for 30 and 60 min detected by western blotting using a specific anti-poly-ADP-ribose antibody (PAR). The *asterisk* denotes a nonspecific band, resulting from a cross-reaction with a component from the serum. (**C**) Poly-ADP-ribosylation levels in RPE-1 wild-type and *HPF1*$^{-/-}$ (clone #5) cells after incubation with 100 µM H_2O_2 on ice in serum-free media for 10 min detected by western blotting using a specific anti-poly-ADP-ribose antibody (PAR). Following lysis, samples were either left untreated or treated with 1 M hydroxylamine for 3 hours at RT. The *asterisk* denotes a nonspecific band, resulting from a cross-reaction with a component from the serum. (**D-E**) Immunofluorescence analysis of ADP-ribosylation levels detected by the specific mono-ADP-ribosylation (MAR 205) binding reagent or the specific poly-ADP-ribosylation (PAR) antibody after detergent-extraction in untreated in U2OS (I) wild-type, *HPF1*$^{-/-}$ (clone I-#5 and I-#8), *PARP1*$^{-/-}$/*PARP2*$^{-/-}$ (clone #5), U2OS (II) wild-type, and *HPF1*$^{-/-}$ (II-cl.1) cells and after treatment with 2 mM H_2O_2 in full media for 20 min at 37 °C. Data represents the mean (±SD) of four to five independent experiments, RFU – relative fluorescence units. Statistical analysis (one-way analysis of variance) is shown (ns – not significant, ***P < 0.001, ****P < 0.0001). The corresponding representative ScanR images for immunofluorescence data are shown. (**F**) Immunofluorescence analysis of ADP-ribosylation levels detected by the specific poly-ADP-ribosylation (PAR) antibody after detergent-extraction in untreated U2OS (I) wild-type, *HPF1*$^{-/-}$ (clone I-#5), U2OS (II) wild-type, and *HPF1*$^{-/-}$ (II-cl.1) cells and after treatment with 0.9 mM MMS for 1 hour at 37 °C. Data represents the mean (±SD) of three independent experiments, RFU – relative fluorescence units. Statistical analysis (one-way analysis of variance) is shown (ns – not significant). The corresponding representative ScanR images for immunofluorescence data are shown.

Supplementary Figure S5. XRCC1 recruitment into chromatin following DNA damage in HPF1-deficient cells (Related to Figure 5). (**A**) RPE-1 wild-type, *HPF1*$^{-/-}$ (clone #5 and #10), and *PARP1*$^{-/-}$/*PARP2*$^{-/-}$ (clone #E6) cells were transiently transfected with mRFP-XRCC1 and microirradiated with a 405 nm UV-laser. Representative images captured pre-irradiation, and at one and five minutes post-irradiation, and quantifications are shown. Data represents the mean percentage (±SD) of transfected cells with XRCC1 recruitment to DNA damage sites, averaged over three independent experiments. Statistical analysis (one-way analysis of variance) is shown (ns – not significant, ****P < 0.0001). A minimum of 5 transfected cells were analysed per sample in each experiment. (**B**) Immunofluorescence analysis of chromatin-bound nuclear XRCC1 after detergent-extraction in U2OS (I) wild-type, *HPF1*$^{-/-}$ (clone I-#5), *XRCC1*$^{-/-}$ (clone #2), U2OS (II) wild-type, and *HPF1*$^{-/-}$ (II-cl.1) cells untreated or treated with indicated doses of H_2O_2 in serum-free media for 10 min on ice. Representative ScanR images and quantifications are shown, RFU - relative fluorescence units. Data are the mean (±SD) of three independent experiments. Statistical analysis (one-way analysis of variance) is shown (ns – not significant, ****P < 0.0001). (**C**) Levels of HPF1 and XRCC1 in whole cell (WC) extracts and chromatin-containing fractionations from U2OS (I) wild-type, and *HPF1*$^{-/-}$ (clone I-#5 and I-#8), and U2OS (II) wild-type, and *HPF1*$^{-/-}$ (II-cl.1) cells before and following treatment with 2 mM H_2O_2 in full media for 20 min at 37 °C. Normalised chromatin bound XRCC1 protein levels, quantified from the western blots, represent the mean (±SD) from three to four independent experiments. Statistical analysis (one-way analysis of variance) is shown (ns – not significant, *P < 0.05).
